# O2-7 Leading the Way Together: A cluster randomised controlled trial of the 5R Shared Leadership Program in older adult walking groups

**DOI:** 10.1093/eurpub/ckac094.015

**Published:** 2022-08-29

**Authors:** Katrien Fransen, Filip Boen, Tegan Cruwys, Catherine Haslam, Peter Iserbyt, Jan Seghers, Julie Vanderlinden, Jannique van Uffelen

**Affiliations:** 1 Movement Sciences, KU Leuven, Leuven, Belgium; 2 Research School of Psychology, The Australian National University, Canberra, Australia; 3 School of Psychology, The University of Queensland, Brisbane, Australia

**Keywords:** walking groups, older adults, peer leaders

## Abstract

**Background:**

With a rapidly ageing society, healthy ageing has become a key challenge for older adults. Engagement in physical activity, and particularly walking, is a key strategy that contributes to healthy ageing. The present study aimed to evaluate the efficacy of a group walking program for older adults that incorporates the 5R Shared Leadership Program (5RS), compared to a regular group walking program. By implementing a structure of shared leadership and strengthening peer leaders' identity leadership, 5RS has in other contexts been associated with greater performance and well-being.

**Methods:**

Our cluster randomised controlled trial included 19 older adult walking groups (i.e., the clusters; N = 503; Mage = 69.23 years, *SD* = 6.68), which all participated in a 12-week structured group walking program. Nine of these walking groups (n = 304) were randomly assigned to the intervention condition and received additionally the 5RS program.

**Results:**

Results revealed that 5RS was successful in strengthening the identity leadership qualities of the appointed peer leaders. Moreover, multilevel regressions showed that 5RS succeeded in increasing group cohesion and walking activity to a greater extent than a regular group walking program, while participants' group identification and well-being increased to a similar extent in both conditions. Furthermore, structural equation modelling revealed that group identification mediated the impact of peer leaders' identity leadership on group cohesion and well-being (but not walking activity).

**Conclusion:**

We can conclude that by harnessing the capacity of the group and its peer leaders, 5RS constitutes a promising intervention to engage older adults in physical activity.

